# Emerging Roles of Mast Cells in the Regulation of Lymphatic Immuno-Physiology

**DOI:** 10.3389/fimmu.2020.01234

**Published:** 2020-06-17

**Authors:** Sarit Pal, Shubhankar Nath, Cynthia J. Meininger, Anatoliy A. Gashev

**Affiliations:** ^1^Department of Medical Physiology, Texas A&M University Health Science Center College of Medicine, Bryan, TX, United States; ^2^Wellman Center for Photomedicine, Harvard Medical School, Massachusetts General Hospital, Boston, MA, United States

**Keywords:** mast cells, lymphatic vessels, lymphatic system, immune response, cancer

## Abstract

Mast cells (MCs) are abundant in almost all vascularized tissues. Furthermore, their anatomical proximity to lymphatic vessels and their ability to synthesize, store and release a large array of inflammatory and vasoactive mediators emphasize their significance in the regulation of the lymphatic vascular functions. As a major secretory cell of the innate immune system, MCs maintain their steady-state granule release under normal physiological conditions; however, the inflammatory response potentiates their ability to synthesize and secrete these mediators. Activation of MCs in response to inflammatory signals can trigger adaptive immune responses by dendritic cell-directed T cell activation. In addition, through the secretion of various mediators, cytokines and growth factors, MCs not only facilitate interaction and migration of immune cells, but also influence lymphatic permeability, contractility, and vascular remodeling as well as immune cell trafficking through the lymphatic vessels. In summary, the consequences of these events directly affect the lymphatic niche, influencing inflammation at multiple levels. In this review, we have summarized the recent advancements in our understanding of the MC biology in the context of the lymphatic vascular system. We have further highlighted the MC-lymphatic interaction axis from the standpoint of the tumor microenvironment.

## Introduction

Since their first description by Paul Ehrlich in 1878 as “mastzellen,” mast cells (MCs) have been mostly viewed as effectors of allergy (1). Investigations from the recent past have shown that nearly all vascularized organs (including heart (2), lungs (3), kidneys (4), intestine (5, 6), and liver (7)), the brain side (8, 9) of the blood-brain barrier, as well as the interfaces of host and the external environment, such as skin (10), have a prevalence of MCs. However, it has only been in the last two decades that MCs have gained significant attention for their involvement in several physiological and pathological processes (11, 12).

## Mast Cell Subsets and Tissue Specificity

MCs are hematopoietic in origin. Following egress from the bone marrow, MC progenitors circulate in the blood, enter various tissues and develop into mature MCs under the influence of local growth factors, such as stem cell factor (SCF) and interleukin 3 (IL3) (13). The hematopoietic development of MC is unique as their early lineage progenitors, known as MC progenitors, leave the bone marrow before they are detectable by any MC lineage-specific histochemical marker and undergo transendothelial migration. Then MCs according to tissue environment differentiate into two major subclasses: (1) connective tissue MCs, located by nerve endings and alongside the blood and lymphatic vasculature, and (2) mucosal MCs, located on mucosal surfaces such as gut and respiratory mucosa. The secretory elements and granule releasing potential of these MC phenotypes are mainly driven by the local factors in these tissue niches (14). In addition, the subpopulations of human mast cells from skin and lungs were initially classified as MC_TC_ (mast cell tryptase and chymase) and MC_T_ (mast cell tryptase) types. They were recognized on the basis of the protease composition of their secretory granules, with tryptase, chymase, carboxypeptidase A3, and cathepsin G in the former and only tryptase in the latter (15, 16). MC_TC_ contribute to tissue remodeling and angiogenesis, whereas MC_T_ have been shown to be associated with host defense and immune functions. Both of these two subtypes express FcεR1 (the high-affinity receptor for the Fc region of immunoglobulin E), enabling them to contribute to allergic and hypersensitivity reactions (17).

The density of MCs in tissues varies depending upon the species and the location. For example, in human skin MC density is 7,000–12,000, 20,000 per mm^3^ in the intestine and 500–4,000 per mm^3^ in the lungs (18). In addition, while MCs are prevalent in lymph nodes, their number is greatly increased in response to inflammation (19), where they actively contribute to the recruitment of immune cells to lymph nodes through secretion of cytokines and chemokines (20, 21).

## Overview of Mechanisms of Mast Cell Activation

**Figure 1:**
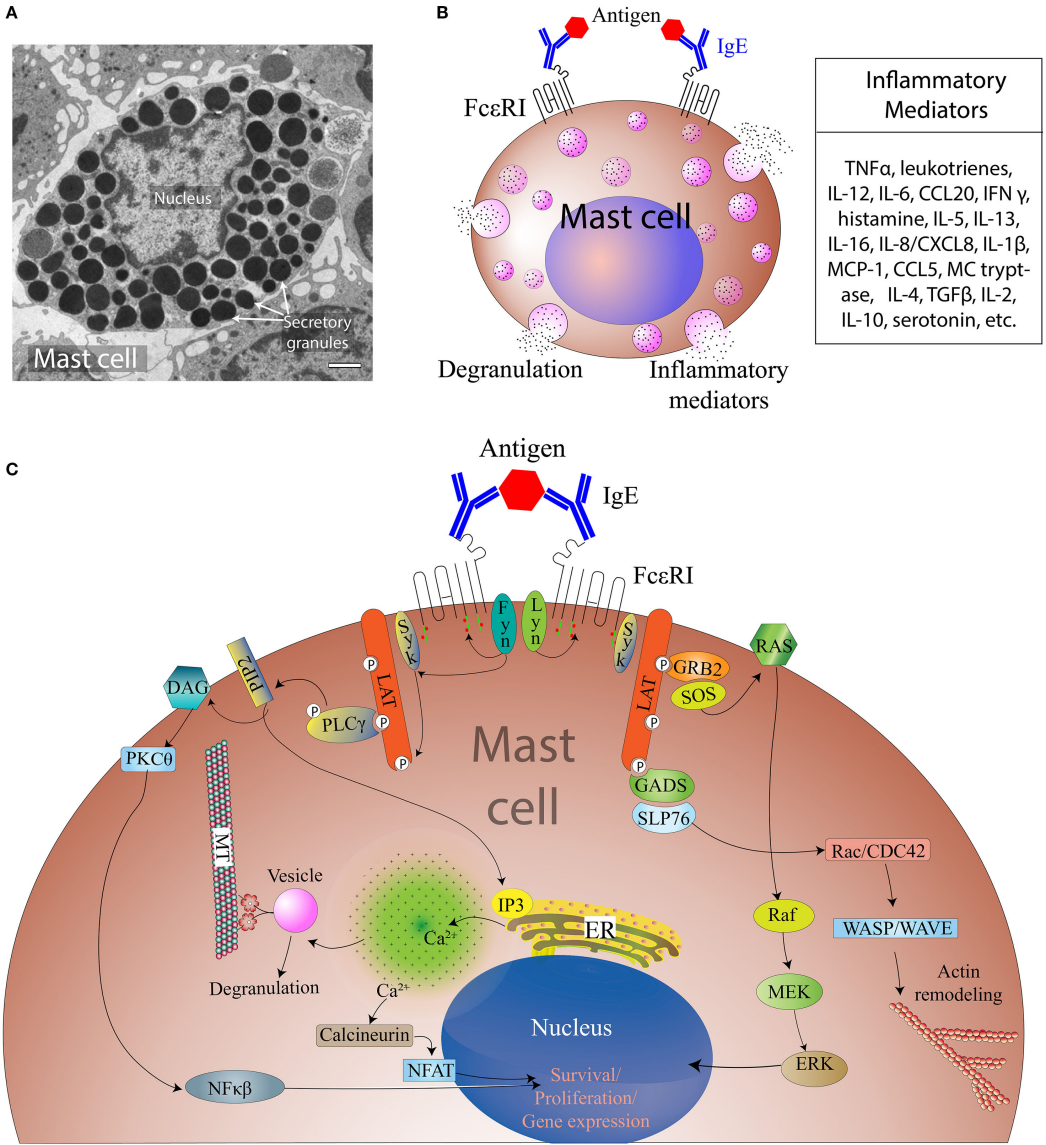
Overview of MC activation and degranulation mechanisms. **(A)** A transmission electron microscope image of an activated MC showing multiple secretory granules inside the cell. Adapted from Grujic et al. (25) and reproduced with written permission from the publisher. Copyright 2013, the American Association of Immunologists, Inc. **(B)** A schematic of a MC showing Immunoglobulin E (IgE)-mediated interaction with allergen and secretion of different inflammatory mediators. **(C)**. Aggregation of the IgE Receptor (FcεRI) by multivalent antigen induces activation of tyrosine-protein kinase Lyn (Lyn), the Src kinase that phosphorylates immunoreceptor tyrosine-based activation motifs (ITAMs) of FcεRI β and γ subunits, followed by the association of the tyrosine-protein kinase Syk with the FcεRI via Syk-Src Homology domain 2 (SH2) within phosphorylated ITAMs. This clustering leads to activation of tyrosine-protein kinase Fyn that phosphorylates the adaptor growth factor receptor-bound protein 2 (Grb2). Activation of phospholipase C gamma 1 (PLC-γ1) results in the hydrolysis of phosphatidylinositol-4,5-bisphosphate (PIP2) into inositol 1, 4, 5-triphosphate (IP3) and diacylglycerol (DAG). IP3 production leads to increased intracellular free calcium (Ca^2+^) concentration, whereas DAG can activate both protein kinase C-θ (PKC-θ) and Ras. Tyrosine phosphorylated SLP76 also associates with the Rho-family guanine nucleotide exchange factor (GEF) Vav1 and the adaptor protein, Nck. Vav1 activates Rac and cell division control protein 42 (Cdc42), which initiate actin cytoskeletal rearrangement via activation of Wiskott-Aldrich syndrome protein (WASP). Cytoskeletal rearrangement is required for cell migration and microtubule-dependent degranulation of MCs.

The cytoplasm of MCs carries secretory granules containing inflammatory mediators (such as histamine), heparin, and a number of cytokines among other mediators (Figures 1A,B). Upon activation, MCs release the contents of these pre-stored granules into their residing tissue microenvironment, initiating multiple physiological responses that are not limited to allergy, but are also involved in control of vascular tone and permeability, neovascularization, and defense against pathogen exposure (22–25) as well as influencing the trafficking of immune cells from the adjacent tissue niche. Interestingly, the recent findings of the close localization and high density of MCs near lymphatic vessels (LVs) and their regulated release of pre-stored as well as *de novo* synthesized vasoactive compounds has expanded the scope of MC biology in the context of lymphatic biology (6, 12, 26–28). Furthermore, recent studies also suggest MCs are immune sentinels, as they are able to present antigens via the expression of major histocompatibility complex II (MHC II) molecules and can regulate the function of innate and adaptive immune cells, including dendritic cells (DCs), macrophages, eosinophils, lymphocytes (T and B cells), and fibroblasts (23, 29–31).

As innate immune cells, MCs are equipped for early and rapid sensing of invading microorganisms such as bacteria, parasites, fungi, and viruses. The magnitude and nature of MC responses to different stimuli can be influenced by intrinsic as well as micro-environmental factors that can modulate the expression and functionality of MC surface receptors and/or signaling molecules contributing to these responses (31, 32). These pathogens display conserved molecular structures called pathogen-associated molecular patterns (PAMPs) that are recognized by pattern recognition receptors (PRRs), such as Toll-like receptors (TLRs), on the MC surface. MCs express TLRs 1 to 7 and 9, NOD-like receptors (NLRs), and retinoic acid-inducible gene-I (RIG-I). Signaling through TLRs on the MC surface activates myeloid differentiation primary response protein 88 (MyD88) and MyD88 adapter like protein/Toll/Interleukin-1 Receptor Domain-Containing Adapter Protein (MAL/TIRAP), which induces nuclear factor kappa-light-chain-enhancer of activated B cells (NF-κB) translocation to the nucleus resulting in the transcriptional initiation of several cytokines. MC-derived histamine is a necessary mediator involved in lipopolysaccharide- (LPS-) induced phosphorylation of NF-κB (33). TLR4 can be activated by LPS, subsequently stimulating MC/histamine/NF-κB-dependent production and release of multiple cytokines by MCs and surrounding tissues (33) as well as the release of preformed granules, whereas activation of TLR2 by peptidoglycan results in extensive degranulation (34, 35). Recent findings demonstrate that histamine, released by MCs, is able to bind to histamine receptors 1 and 2 on MCs and, as such, maintains or re-initiates further MC degranulation (12).

The most extensively investigated pathway for MC activation (schematically presented in Figure 1C) is mediated through antigen/IgE/FcϵRI cross-linking. The high affinity immunoglobulin E (IgE) receptor, FcϵRI, consists of an α-chain that binds to IgE, a β-chain that spans the cell membrane, and two γ chains. Tyrosine-protein kinase Lyn (Lyn) interacts and phosphorylates tyrosine in its immunoreceptor tyrosine-based activation motifs (ITAMs) on the β and γ chains of the FcϵRI, which further activates Syk tyrosine kinases that phosphorylate LAT1 and LAT2 (linkers for activation of T cells). Furthermore, downstream phosphorylated phospholipase Cγ1 (PLCγ1) hydrolyzes phosphatidylinositol-4,5-bisphosphate (PIP2) to make inositol-1,4,5-trisphosphate (IP3) and diacylglycerol (DAG), causing calcium (Ca^2+^) mobilization from the endoplasmic reticulum. The release of Ca^2+^ from the endoplasmic reticulum leads to stromal interaction molecule 1- (STIM1) mediated opening of the store-operated Ca^2+^ channel Orai1, leading to the influx of extracellular Ca^2+^. The influx of Ca^2+^ is accompanied by an additional mechanism that is mediated by transient receptor potential channel 1 (TRPC1). The increase in intracellular Ca^2+^ levels and the activation of PKC triggers the degranulation machinery. Calcium release also activates NF-κB translocation to the nucleus, which results in transcriptional initiation of several cytokines, such as IL6, tumor necrosis factor alpha (TNFα), and IL13. However, activation of FcϵRI also activates Fyn (Src kinase), complementary to the Lyn signaling pathway, which can also modulate MC degranulation. Fyn activates Syk which in a downstream cascade activates mammalian target of rapamycin (mTOR) in an Akt-dependent manner, and this can also induce MC chemotaxis and granule release (25, 36–39).

In addition to these mechanisms, MCs can be activated by a wide range of stimuli such as neuropeptides, cytokines, growth factors, vasoactive mediators such as non-MC-derived lymphatic-derived (40, 41) histamine, leukotrienes, toxins, certain lectins, basic compounds, complement proteins, immune complexes, certain drugs, as well as physical or mechanical stress and stretch. Recent investigations have shown that receptors for adenosine, complement C3A, chemokines, sphingosine-1-phosphate, substance P and SCF are also involved in MC activation (42–45). Activation of these receptors can also potentiate FcεRI-mediated activation (42–45).

An important aspect of the regulation of MC granule release is MC sensitization. When the host is exposed to pathogens, antigen-presenting cells are engaged. Subsequent activation of Th2 cells along with interleukin production causes B cells to undergo class-switching to form IgE antibody secreting plasma cells. IgE directly binds to the high affinity receptor FcεR1 expressed on MCs. In the process of B cell differentiation to IgE-secreting plasma cells, the MCs secrete interleukins-4,−5, and−10, all of which are crucial for this process. Thus, a MC-dependent MC sensitization loop can aggravate inflammatory pathophysiology. The precise mechanism by which this MC feedback regulation can be disrupted without inducing pathological outcomes is still not clearly understood, however repeated exposure to increasing doses of antigen or oral immune therapy (46) can potentially ameliorate or reduce the degree of MC sensitization (47) and thereby associated pathology.

## Mast Cells as Effector Cells at the Interface of the Lymphatic and Immune Systems

Beyond the role of MCs in hypersensitivity reactions, as an immune sentinel MCs respond to a variety of pathogenic (and non-pathogenic) stimuli by releasing a host of vasoactive mediators, cytokines, growth factors, proteases, biogenic amines and interferon pre-stored within MC granules (see Table 1) (85). Release of granules can be broadly divided into two types: (1) degranulation, characterized by rapid release of pre-synthesized granules [often seen in allergic reactions], and (2) *de novo* mediator release, a comparatively slow process contributing to chronic responses in tissue remodeling, pathogen clearance and often involving engagement of innate as well as adaptive immune cells. MCs, as innate immune sentinels, preferentially reside in the interface of the host and its external environment (36, 86). Exposure of MCs to Gram-negative bacteria induces release of inflammatory mediators such as TNFα, IL6, and IL1β, and chemotactic migration of natural killer cells, eosinophils, and neutrophils, as well as upregulation of adhesion molecules and chemokine ligands in blood and lymphatic vasculatures (26). Activation of MCs also modulates both adaptive and innate immune responses (85, 87). For example, release of histamine by MCs has been shown to regulate T helper type 1 (Th1) and Th2 responses by inducing Th1-specific (IL4, IL10, IL13) and Th2-specific (IFNγ, IL2) (88) cytokines through differential activation of histamine receptors 1 and 2 (61). A recent study by Kambayashi et al. demonstrated that in certain inflammatory conditions MCs may express MHC II molecules and present antigens to CD4+ T cells and preferentially expand T regulatory cells (89). Furthermore, studies on the formation of MC-DC (12, 90) or MC-T cell (91) immune synapses reinforce the possibility of intercellular crosstalk and ability of MCs to recruit DCs or T cells to the site of inflammation, infection or injury. As such, MCs are endowed with the ability to not only modulate innate but also adaptive immune responses (28, 92). In addition, recent evidence suggests that MCs can increase their IL10 secretion in a T regulatory cell-dependent manner. This, in turn, contributes to the maintenance of graft tolerance (93). Therefore, while T cells require MHC-bound antigens on antigen-presenting cells to trigger T cell secretory pathways (94), MCs do not require any MHC-bound antigen to initiate the degranulation process. However, aberrant activation of MCs can cause MC degranulation, which can suppress T regulatory cell function, which essentially breaks down peripheral tolerance (95).

**Table 1:** MC-secreted major mediators in immune regulation.

**Mediators**	**Types of immune cell involved**	**Functions**	**References**
TNF-alpha	Naive T cells Effector T cells Macrophages	Activation and proliferation of naive and effector T cells and suppression of T regulatory cell (Treg) activation, activation of macrophages	(48, 49)
Leukotrienes (LTB4, LTC4)	Neutrophils DCs	Recruitment and chemotaxis	(50–52)
IL12	Th1 cells	Initiation of a Th1 type response	(53, 54)
IL6	Neutrophils MCs Macrophages T helper cells	Regulation of inflammatory reactions, chemotaxis, macrophage M2 polarization, T helper cell polarization	(55–57)
CCL20	DCs	Recruitment to inflammatory site	(58, 59)
IFN Gamma	Th1 cells	Th1 response, migration and proliferation	(60)
Histamine	Th1 cells DCs	Cellular differentiation, chemotaxis	(61, 62)
Serotonin	T cells Monocytes Macrophages	Chemotaxis, proliferation, cytokine secretion	(63–65)
IL5	B cells	Terminal differentiation of activated B cells	(66, 67)
IL13	Fibroblasts	Facilitation of a Th2 type response	(67)
IL16	CD4+ T cells	T cell growth and chemotaxis	(68)
IL8 (CXCL8)	Neutrophils	Chemotaxis	(69)
IL1-beta	DCs and MCs	T cell-independent DC activation IL8 synthesis	(70–72)
MCP-1 (CCL2)	DCs, memory T lymphocytes, macrophages	Recruitment	(73, 74)
MC chymase	Neutrophils	Recruitment	(75)
RANTES/CCL5	Th2 cells	Polarization toward Th2 phenotype	(76)
MC tryptase	Neutrophils	Recruitment	(77)
IL4	Th2 cells B cells	Differentiation of naïve T cells to Th2 cells, migration of T cells and B cells B cell maturation, B cell survival signal	(67, 78, 79)
TGF beta	iTregs, Th2 cells, B cells	Development of T regulatory cells (Tregs), B cell apoptosis and maturation	(80, 81)
IL2	Th1 cells Tregs	Th1 and 2 differentiation, Treg survival and development	(82, 83)
IL10	T follicular helper cells	Downregulation of Th1 cytokines	(84)

**Figure 2:**
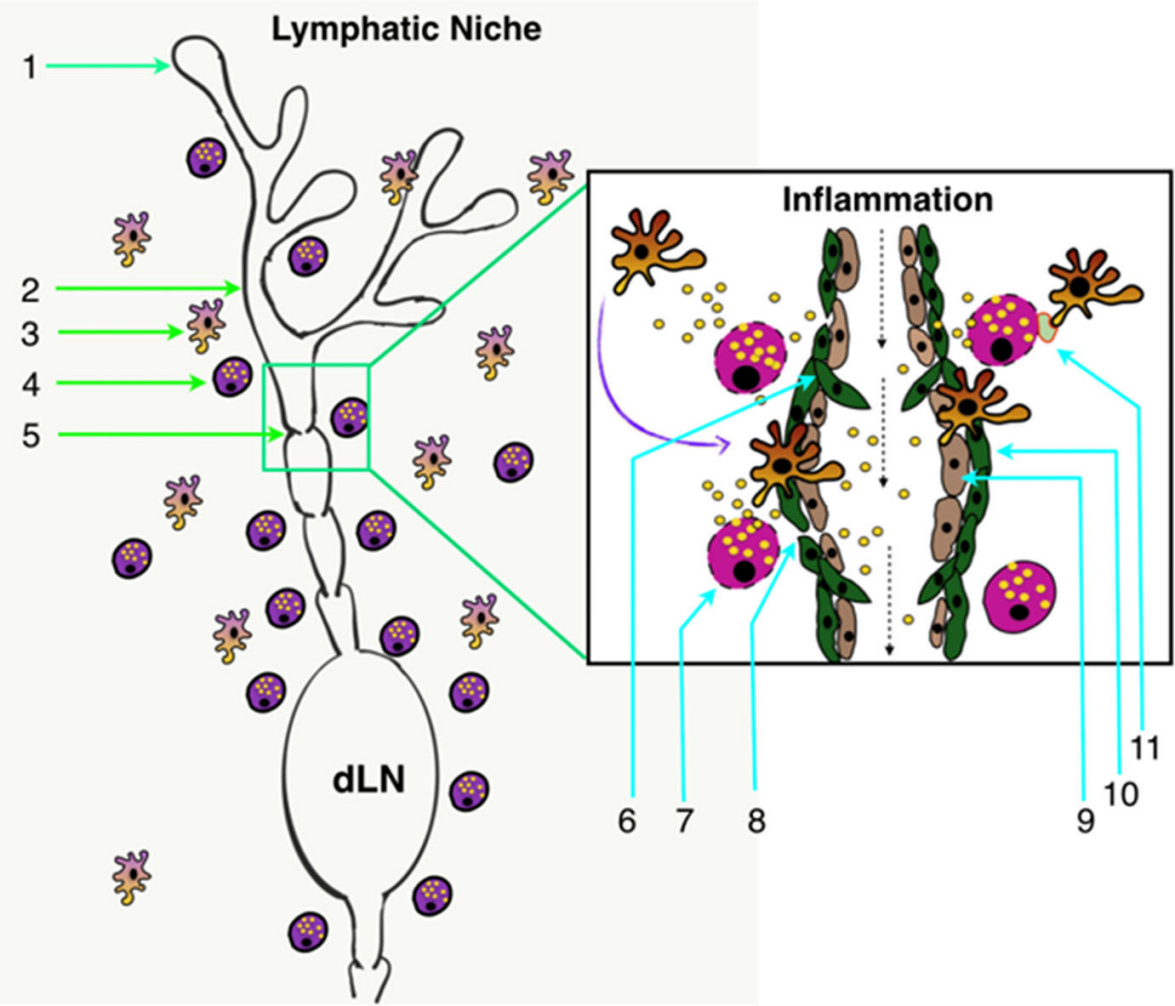
Lymphatic system architecture along with the lymphatic tissue niche. (1) Initial lymphatics (lymphatic capillaries) (2) Pre-collector LVs; (3) Perilymphatic antigen-presenting cells; (4) Perilymphatic MCs; (5) Collecting LVs; (6) Lymphatic valve in collecting LVs; (7) Degranulating MCs in response to inflammation; (8) Increased permeability of LVs in response to inflammation; (9) LECs; (10) Lymphatic muscle cells; and (11) Inflammation-induced MC-DC immune synapse formation in perilymphatic tissues. dLN, Draining lymph node.

MCs are the major innate effector cell type localized close to LVs (6, 12, 26, 28) (Figure 2) and are able to release numerous inflammatory and vasoactive mediators (Tables 1, 2). These unique features make MCs a relevant and key player in the regulation of lymphatic immuno-physiology. The functional implication of such a lymphatic-oriented localization pattern of MCs is not completely understood. However, we believe that it is reasonable to speculate that as the major sensory arm of the innate immune response, activated and degranulated MCs can release a wide array of vascular and inflammatory mediators (135) that are able to diffuse to the adjacent LVs due to their proximity (see Table 2). These mediators can instantaneously interact with the lymphatic endothelium and promote expression of adhesion molecules, such as integrins, that promote the recruitment of circulating leukocytes as well as affecting the permeability of LVs (discussed in details in (33)). Furthermore, chemotactic migration of leukocytes from the tissue parenchyma facilitates LV-directed immune cell trafficking, advancing the resolution of a local inflammatory event (12, 28). MC mediators also influence lymphatic pumping by affecting lymphatic muscle cell contractility (33, 96), thus accelerating or slowing down delivery of pathogens and immune cells to draining lymph nodes. Many of these events are dysregulated due to age-related alterations in LVs and signaling pathways that lead to MC degranulation. In brief, aging alters structure (by increasing the size of zones with low muscle cell investiture) (136), ultrastructure (through loss of the glycocalyx), and proteome composition with a concomitant increase in permeability of aged lymphatic vessels (137). The contractile function of aged LVs is depleted (138–140) with the abolished role of nitric oxide and an increased role of lymphatic-born histamine in flow-dependent regulation of lymphatic phasic contractions and tone (40, 41). In addition, aging induces oxidative stress in LVs (141) and facilitates the spread of pathogens from these vessels into perilymphatic tissues (137). Aging causes the basal activation of perilymphatic MCs, which, in turn, restricts recruitment/activation of immune cells in perilymphatic tissues (6, 28). This aging-associated basal activation of MCs limits proper functioning of the MC/histamine/NF-κB axis that is essential for the regulation of LV transport and barrier functions as well as for both the interaction and trafficking of immune cells near and within lymphatic collecting vessels (33). Cumulatively, these aging-associated changes in MCs play important roles in the pathogenesis of alterations in inflammation and immunity associated with aging (33, 142, 143).

**Table 2:** MC-derived Mediators that Regulate the Lymphatic Vasculature.

**MC mediators**	**Affected lymphatic functions**	**Other major effects/targeted signaling mechanisms**	**References**
Histamine	Lymphatic contractility, lymphatic permeability, immune cell recruitment to LVs	PKC/ROCK/NO HR/NF-κB ROCK, cAMP	(12, 28, 96–110)
Leukotrienes (LTB4, LTC4)	Lymphatic permeability, immune cell recruitment to LVs	P-Selectin	(98, 111)
Serotonin	Lymphatic contractility	cAMP/cGMP	(112)
TNF-alpha	Expression of adhesion molecules in lymphatic endothelial cells (LECs), lymphangiogenesis, lymphatic pumping	Endothelial leukocyte adhesion molecule-1, intercellular adhesion molecule-1, vascular cell adhesion moleculeNF-κB, iNOS	(113–116)
IL6	Inflammation-induced lymphangiogenesis	Src-MAPK-VEGF-c in LECs, upregulation of adhesion molecules in LECs	(117)
VEGF	Lymphangiogenesis, lymphatic permeability	PI3K-HIF-VEGF	(26, 118, 119)
Bradykinin	Lymphatic contractility, lymphatic permeability	Kinin B2 receptor j-dependent manner	(120, 121)
IL8	LEC proliferation, immune cell recruitment to LVs	Adhesion molecule expression on LECs	(122, 123)
MC tryptase	Immune cell recruitment to LVs, matrix degradation	CCL2, IL8 expression on LECs	(26, 124, 125)
MC chymase	Matrix remodeling, neutrophil recruitment	Conversion of Ang I to Ang II, activation of pro-MMP 9	(124, 126)
Prostaglandin E2	Lymphatic contractility	PKA-dependent actions	(103, 127)
PDGF	Lymphatic contractility, lymphangiogenesis	NO-mediated MAPK activation	(128–130)
FGF	Lymphangiogenesis	Activation of VEGF-C and VEGF-D	(131, 132)
IFN gamma	Lymphatic permeability, lymphocyte binding with LECs	Endothelin-1 and VE-Cadherin	(133, 134)

## Role of Lymphatic Vasculature-Associated Mast Cells in the Regulation of the Inflammatory Response

Local inflammation causes pathological outcome such as swelling, where chemotactic migration of leukocytes into the affected area is accompanied by the rapid influx of interstitial fluid. Such pathophysiological change increases the overall tissue interstitial pressure gradient. This pulls on the anchoring filaments attached to lymphatic capillaries (initial lymphatics), opening primary lymphatic valves and helping various immune cells to enter lymphatic network, to clear accumulated antigens and noxious tissue debris from the inflamed site (144–147). The phasic contractions of lymphangions in LVs support long-distance flow of lymph and immune cells and direct the immune cell trafficking from the affected site to the draining lymph nodes (146, 147). Furthermore, lymph nodes, as secondary lymphoid organs, are continuously perfused and presented with soluble foreign antigens by the lymph transported through afferent LVs. Continuous screening of lymph in the node is necessary for activation of node-resident naïve antigen-presenting cells and priming of naïve T and B cells in response to any given inflammatory stimuli.

In addition, novel studies suggest that inflammation-induced changes to the microenvironment can upregulate the expression of adhesion molecules such as ICAM-1 and VCAM-1, as well as chemokines such as CCL21 and CX3CL1 in lymphatic endothelial cells (LECs) (148, 149). This upregulation can enhance trafficking of antigen-presenting cells, such as DCs and macrophages, from the affected tissues through the walls of collecting LVs toward the draining lymph node, thus contributing to the resolution of tissue inflammation (145, 146, 150). In addition, molecules such as the chemokine scavenging receptor D6 expressed by LECs can selectively regulate the interaction between mature and immature DCs, which helps in the cellular trafficking and removal of inflammatory chemokines. At the same time, the expression of D6 is dependent on molecules such as IL6 and interferon gamma secreted by the MCs and present in the inflammatory microenvironment (151). In addition, inflammation can induce expansion of the lymphatic network through initiation of lymphangiogenic programs, driven by vascular endothelial growth factor-C (VEGF-C-) and VEGF-D-related signaling pathways. Their activation depends on molecules such as TNFα, fibroblast growth factor (FGF), platelet-derived growth factor (PDGF) and IL6 secreted by immune cells closely associated with LVs, such as perilymphatic MCs (Table 2), as well as tissue-resident or migratory macrophages. Although considerable progress has been made in understanding the basic mechanisms of lymphangiogenic programs, the question on how these regulatory factors induce inflammation-associated lymphangiogenesis warrants further investigation (152).

It has been suggested that lymphangiogenesis contributes to chronic inflammation-associated pathology or to the resolution and repair of damaged tissue. However, a chronic inflammatory environment can also influence not only lymphatic muscle and endothelial cells but also activate regulated release of granules by MCs. Overall, an inflammation-induced, MC-dependent dysfunction of LVs (33) may cause significant local stasis of lymph as well as diminished trafficking of lymphocytes to the draining lymph node causing overall immune suppression. The resultant altered tissue environment is manifested clinically, e.g., as lymphedema, local fibrosis, and secondary bacterial infections. These pathologies are strongly associated with diseases such as secondary lymphedema, often occurring following the surgical removal of lymph nodes to limit cancer progression or in cases of lymphatic filariasis or lipedema (153).

## Roles of Mast Cells in the Modulation of Lymphatic Immuno-Physiology in Cancer

Genetic and epigenetic alteration of healthy as well as tumor-associated cells play a pivotal role in the development of tumor microenvironment and progression of tumor pathology. The presence of MCs in human tumors was known for more than a century (154), however their specific roles in cancer have just begun to be explored in recent decades. Several studies have shown an increased number of MCs in tumors, such as squamous cell carcinoma of the esophagus (155), pancreatic adenocarcinomas (156–158), prostate cancer (159), breast cancer (160, 161), and many hematological carcinomas (162–164). The studies on MCs in tumor sites suggest a potential role of MCs in tumor progression (165, 166). However, whether MCs play an anti-tumorigenic or pro-tumorigenic role remains controversial (165, 167) and has become an active area of research.

**Figure 3:**
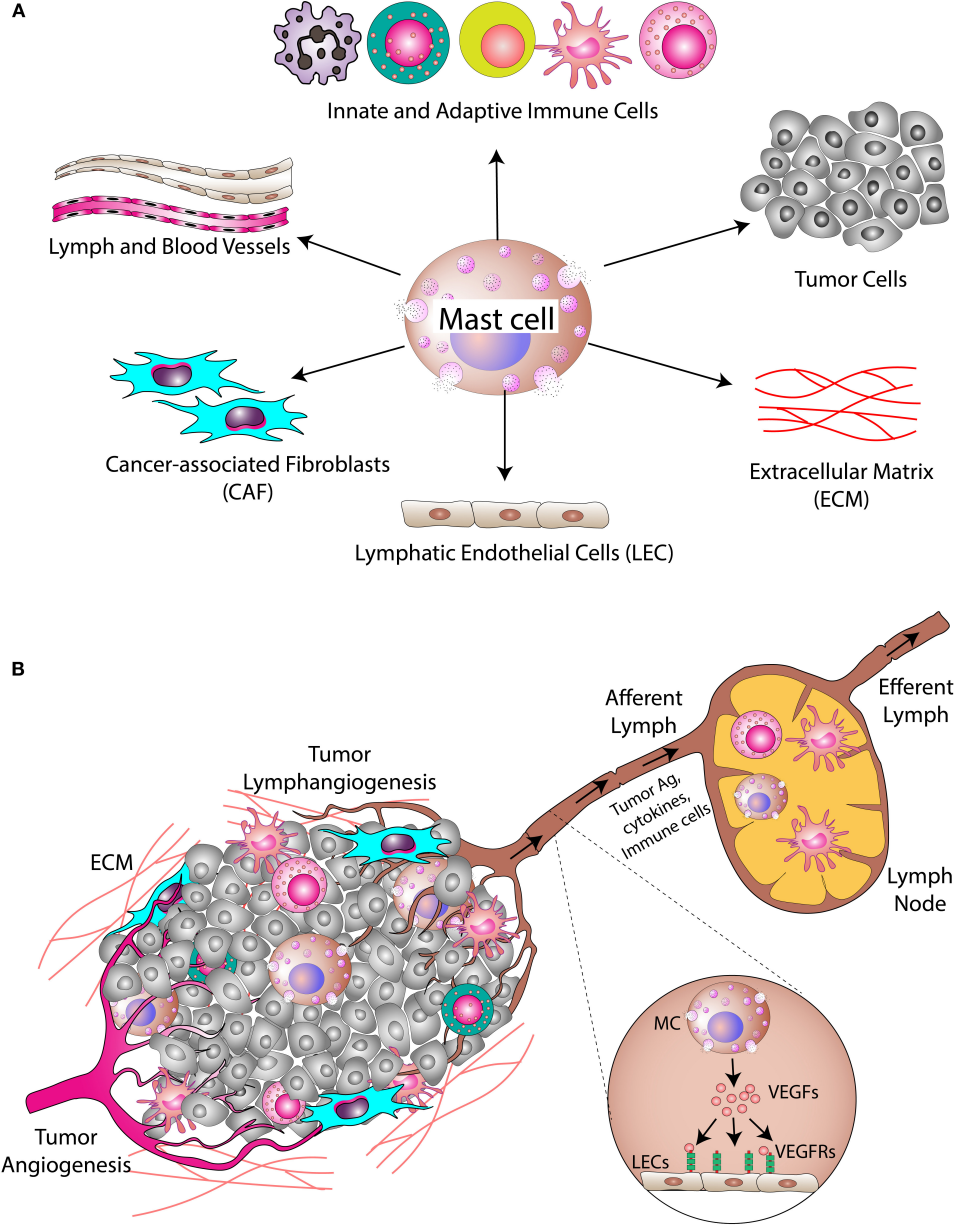
Mast cells in the tumor microenvironment. **(A)** MCs secrete different cytokines/inflammatory mediators into the tumor microenvironment. These cytokines/inflammatory mediators modulate cellular and acellular components of the tumor. The cellular components include cancer cells, cancer-associated fibroblasts, and different types of immune cells, while the acellular component is mainly the extracellular matrix (ECM). **(B)** MCs also modulate angiogenesis and lymphangiogenesis in the tumor. Tumor necrosis factor-alpha (TNFα) released by MCs induces migration of DCs into the draining lymph nodes where T cell activation takes place. MC-derived proteases induce modification of the ECM, which alters the microarchitecture leading to metastasis.

The cross-talk between MCs and other cells such as cancer-associated fibroblasts (CAFs), tumor cells, myeloid-derived suppressor cells, and immune cells in the tumor microenvironment is being explored by many research groups. MCs secrete several cytokines, depending on their activation status, which directly and indirectly impact the behavior of cancer cells and other components of the tumor microenvironment (Figure 3A). TNFα released by MCs helps DCs to mature and express more MHC Class I molecules and co-stimulatory receptors on MCs that activate CD8+ T cells in the tumor-draining lymph node (168, 169). Since activated CD8+ T cells play a critical role in eliminating cancerous cells from the body, it is important to explore to what extent MC-mediated activation of T cells contributes to cancer immunity (170). In one study, MCs were shown to be present in metastatic lymph nodes of cancer patients (171), suggesting a connection between the immune and lymphatic system in cancer. Interestingly, Stelekati et al. showed that MCs are capable of presenting antigens in the context of MHC class I and II molecules (168). In this latter study, MC-mediated antigen presentation was found to regulate cytotoxic T lymphocyte effector function. Another study by Nakae et al. reported that MCs express several co-stimulatory molecules and can activate T cells in a TNFα-dependent manner (169). MCs were also reported to release several cytokines that direct the functions of a subset of T cells (82). Thus, like other professional antigen presenting cells, MCs are also capable of providing signals to T cells: antigen presentation, expression of co-stimulatory molecules, and release of cytokines. However, these events happen in different contexts and in a poorly coordinated manner, and are not necessarily related to cancers. Antigen presentation by MCs has also been reported in the case of bacterial infections (172). Taken together, these findings broaden our understanding of the capabilities of MCs to serve as non-professional antigen presenting cells.

SCF is among the many inflammatory mediators produced and secreted by tumor cells in the tumor microenvironment. SCF is known to bind the c-kit receptor on the surface of MCs (43, 173). Activation of this signaling cascade is necessary for maturation, migration, and survival of MCs, which could explain why an increased accumulation of MCs is observed in the tumor microenvironment of many cancers. A study led by Zhang et al. (174) provided direct evidence that SCF released by tumor cells modulates tumor angiogenesis by regulating MCs. In this study, the researchers used sense or antisense SCF cDNA to overexpress or deplete SCF expression in rat mammary tumor cells. Depletion of SCF significantly decreased MC infiltration and vascularization in the tumor whereas the opposite effects were observed in SCF-overexpressing tumors. Thus, SCF should be considered as a therapeutic target to inhibit the progression of certain types of tumors. A later study by Huang et al. (175) suggested a role for SCF-activated MCs in remodeling of the tumor microenvironment and subsequent immunosuppression. The activated MCs were shown to release adenosine, which in turn increased T regulatory cell infiltration into the tumors.

Angiogenesis and lymphangiogenesis are very critical steps in the development of a tumor since their induction is necessary for the nourishment of growing tumors (176, 177). In addition, the tumor-associated lymphatic network also plays a pivotal role in the process of metastasis (178). Studies have shown that cancer cells leave their site of origin and migrate to tumor-draining lymph nodes (179). The presence of cancer cells in tumor-draining lymph nodes serves as a major prognostic indicator in many cancers, including breast, skin, and colon cancers. Thus, the lymphatic network within and around a solid tumor serves as a route for dissemination and metastasis of cancer cells (Figure 3B).

The evidence that MCs are involved in lymphangiogenesis comes from multiples studies. In a report by Raica et al. (180), MCs were identified as the key player in the development of tumor LVs in a certain subtype of breast cancer. The study was conducted by analyzing histopathological samples from human patients that showed a positive correlation between peritumoral MC density and lymphatic microvessel density. In contrast, Utrera-Barillas et al. (181) reported a direct correlation between MC density and blood vasculature, whereas macrophage density was directly correlated with lymphatic vasculature in certain stages of cervical carcinoma, suggesting tumor type-specific roles for MCs in neovascularization.

Poor prognosis, invasion, and metastasis of cancer are often associated with increased LV density and secretion of VEGF-C (182–184), a pro-lymphangiogenic cytokine known to be secreted by many cancer cells (182, 185, 186) as well as MCs (118). VEGF-C increases the rate of lymph flow to the tumor-draining lymph node (187), which could facilitate cancer metastasis. However, further studies are warranted to draw a concrete link between them. Tumor cells can also gain metastatic features as a result of epithelial-to-mesenchymal transition (EMT). EMT is associated with chemoresistance in many cancer types. MCs were reported to induce EMT and cancer stem cell signatures in human thyroid cancer through CXCL8/IL-8 pathways (188, 189).

MCs can remodel the tumor microenvironment not only by regulating the cells but also by changing the tumor matrix, commonly known as extracellular matrix (ECM). MCs secrete different matrix metalloproteinases (such as MMP9) (190) and proteases, including tryptase and chymase (77, 191). These enzymes digest tumor matrix favoring the expansion and migration of tumor and other cells in the microenvironment. Such matrix degradation also disrupts the physical contact between epithelial and stromal layers, leading to detachment of tumor cells and metastasis (192, 193).

In summary, the involvement of MCs in tumor progression is multi-faceted. Several studies of different cancers in human patients and experimental models indicate that MCs promote tumor growth by suppressing immunity and/or by promoting angiogenesis, while other studies indicate MCs are instrumental in inhibiting tumor growth. These studies have been summarized and discussed in multiple recent review articles (165, 167, 194). While the controversy remains as to whether MCs act as pro-tumorigenic or anti-tumorigenic cells, it is probably safe to assume that their functions are tumor type-specific in nature and cannot be generalized. Furthermore, it has long been debated whether inflammation is linked to cancer (195, 196) and to what extent MCs are involved in this process in order to develop new treatment lines and preventive measures. The precise mechanism by which MC-mediated inflammation leads to cancer progression, if any, is yet to be determined.

## Conclusions and Perspectives

For many years, MCs have only been perceived as an integral part of the innate immune system. However, recent studies on MCs have tremendously changed our understanding of their roles in the development of adaptive immune responses as well as their close association with lymphatic vasculature. Evidence presented in this review suggests that MCs are intricately involved in the regulation of lymphatic functions, thus contributing to the convergence of the immune system with the lymphatic vascular system. Through the secretion of various mediators, cytokines and growth factors, MCs not only facilitate cellular interaction and migration but also influence lymphatic permeability, contractility, and vascular remodeling and immune cell trafficking toward and through the LVs. These MC-mediated physiological alterations on LVs are also critical to cancer progression. The signaling axis and the cross-talk between MCs and different cell types in the lymphatic niche of healthy and cancerous tissue remain poorly defined. Certainly, this lymphatic-MC-adaptive immune axis is greatly dependent upon the various inflammatory contexts as well as a tissue- or tumor-specific microenvironment. However, many of these pathways and the crosstalk between MCs and other cells have yet to be explored, warranting further studies to understand many diseases associated with inflammation and to determine further in depth how MCs could be approached as a potential therapeutic target for next-generation medicine.

## Author Contributions

All authors contributed to manuscript writing, read, and approved the submitted version.

## Conflict of Interest

The authors declare that the research was conducted in the absence of any commercial or financial relationships that could be construed as a potential conflict of interest.
